# Ginsenoside Rg3 targets glycosylation of PD-L1 to enhance anti-tumor immunity in non-small cell lung cancer

**DOI:** 10.3389/fimmu.2024.1434078

**Published:** 2024-08-23

**Authors:** Wei Wang, Min Kong, Fu Shen, Ping Li, Cheng Chen, Yueqin Li, Cheng Li, Zhiqiang Qian, Aihua Zhong, Yuhua Wang, Liang Yang, Fangkai He, Weichun Li

**Affiliations:** ^1^ Taizhou Jiangyan Traditional Chinese Medicine Hospital, Jiangyan Affiliated Hospital of Nanjing University of Chinese Medicine, Taizhou, Jiangsu, China; ^2^ Department of Pharmacy, The Affiliated Taizhou People’s Hospital of Nanjing Medical University, Taizhou, Jiangsu, China; ^3^ Department of Radiology, Shanghai Changhai Hospital, Shanghai, China; ^4^ Department of Respiratory and Critical Care Medicine, Kunshan Hospital of Traditional Chinese Medicine, Kunshan, Jiangsu, China

**Keywords:** ginsenoside Rg3, non-small cell lung cancer, PD-L1, glycosylation, T cell activation

## Abstract

**Background:**

Reactivate the T cell immunity by PD-1/PD-L1 checkpoint blockade is widely used in non-small cell lung cancer (NSCLC) patients, while the post-translational modification of Programmed death ligand-1 (PD-L1) is commonly existed in various cancer cells, thus increases the complexity and difficulty in therapy development. Ginsenoside Rg3 is an active component of traditional Chinese herb Ginseng with multiple pharmacological effects including immune regulation. However, the effect on the glycosylation of PD-L1 is unknown.

**Methods:**

NSCLC cell lines were tested for glycosylation of PD-L1, and the potential mechanisms were investigated. Tumor cell-T cell coculture experiment was conducted and the activation of T cells and cytotoxicity were measured by flow cytometry. In vivo xenograft mouse tumor model was used to investigate the effects of Rg3 on PD-L1-mediated immunosuppression and tumor growth.

**Results:**

Here, we identified PD-L1 is widely N-linked glycosylated in NSCLC cell lines, while Rg3 could inhibit the glycosylation of PD-L1 by downregulating the EGFR signaling and further activate GSK3b-mediated degradation, thus resulted in reduced PD-L1 expression. Moreover, the inhibition of PD-L1 glycosylation promoted the activation and cytotoxicity of T cells under coculture condition. In addition, Rg3 could decrease the tumor volume and enhance anti-tumor T cell immunity as evidence by the upregulated expression of Granzyme B and perforin in CD8+T cells, along with elevated serum IL-2, IFN-g and TNF-a level in Rg3-treated mice.

**Conclusions:**

These results suggest that Rg3 inhibits PD-L1 glycosylation and thus enhance anti-tumor immunity, which provide new therapeutic insight into drug discovery.

## 1 Introduction

Lung cancer is the most common cause of cancer death around the world, and nearly 85% of lung cancer patients are identified as non-small cell lung cancer (NSCLC), including lung squamous cell carcinoma and lung adenocarcinoma (1, 2). Lung cancer is a heterogeneous disease and full of complexity, thus understanding the underlying mechanisms that govern the malignant progression is of great importance to achieve better patient outcomes. The most common genetic alterations in NSCLC are Kirsten rat sarcoma (KRAS) and epidermal growth factor receptor (EGFR) genes, which contribute to the resistance of certain targeted inhibitors (3, 4).

With the development of immunotherapy, the treatment of lung cancer has changed from the use of cytotoxic therapy to a serial of targeted therapies or immunotherapies, such as immune checkpoint blockers like monoclonal antibody-based drugs targeting programmed cell death protein-1 (PD-1) or programmed death ligand-1 (PD-L1). Nivolumab, pembrolizumab and atezolizumab have been used as a standard therapy in clinical treatment of NSCLC (5–7). Despite PD-1/PD-L1 blockade therapy has shown remarkable clinical benefits, response rates are still not so high due to the expression of PD-L1 and infiltration of T cells (8). PD-L1 is a 33 kDa transmembrane protein that binds to PD-1 to inhibit T cell proliferation and function (9, 10). PD-L1 is widely expressed in various cell types, including epithelial cells, endothelial cells, macrophages and neutrophils. The upregulation of PD-L1 in NSCLC is well-characterized, and the typical mechanisms of PD-L1 upregulation mainly include JAK-STAT-IRF1/TLR4/MAPK/PI3K signaling to transcriptionally upregulate PD-L1 (10, 11). However, recent studies have identified serval post-translational modifications of PD-L1 to regulate the stability and function of PD-L1, including phosphorylation, glycosylation and polyubiquitination (12).

Among these, the glycosylation of PD-L1 is of great importance to regulate the stability of PD-L1 and the interaction of PD-1. PD-L1 is commonly *N*-linked glycosylated at N35, N192, N200 and N219 (13). Once the PD-L1 is glycosylated, it protects PD-L1 from degradation, thus promoting the stability of protein. In fact, the non-glycosylated form of PD-L1 undergo rapid protein degradation in cells, which provides the new therapeutic strategy for clinical management. Interestingly, serval studies have demonstrated that PD-L1 is highly glycosylated in tumors, including breast cancer, hepatocellular carcinoma and melanoma (13–16). Moreover, Lee and colleagues identified *N*-linked glycosylation of PD-L1 hinders the recognition by antibodies, thus partially explains the different outcome between patients when using the same checkpoint blockade therapy (14). Thus, targeting glycosylation of PD-L1 may provide new insight into immune-based anti-tumor therapies.

Ginsenoside Rg3 is the active component in ginsenosides, which exerts multiple pharmacological effects including anti-inflammation, anti-tumor, and anti-infection (17–19). Notably, Rg3 have been recognized to enhance the treatment of NSCLC, including gefitinib and icotinib (20). Rg3 could also enhance anti-tumor immunity, while the underlying mechanism remains unclear. Thus, we hypothesized that Rg3 might have impact on the regulation of PD-L1 glycosylation. In the present study, we first confirmed the glycosylation of PD-L1 in NSCLC and evaluated the effect of Rg3. We next uncovered EGFR-GSK3β signaling axis was involved in the inhibition of PD-L1 glycosylation and demonstrated the enhanced T cell activation upon treatment of Rg3. Using mouse model of lung cancer, we further confirmed the pharmacological effects of Rg3, along with enhanced CD8+T cell activation and cytotoxicity. Taken together, our research unraveled the new application and new mechanism of Rg3 in treating NSCLC.

## 2 Methods

### 2.1 Animals

6-week-old C57/BL6J nju male mice were procured from GemPharmatech Co., Ltd (Nanjing, China). Mice were housed under standard laboratory conditions (room temperature: (22 ± 2°C); humidity: (50 ± 5) %) with a light/dark cycle of 12/12 h (lighting on at 7:00 a.m.). All experimental protocols were approved by the Animal Care and Use Committee of Nanjing University of Chinese Medicine (Nanjing, China) and conducted conforming to the Guidelines for the Care and Use of Laboratory Animals (ACU170906).

### 2.2 Mouse models of lung cancer

The mouse xenograft model of lung cancer was established via injection of 2×10^6 LLC cells were injected into the right flank. Body weight and tumor size were measured every 3 days after the tumor volume reached 100 mm^3^. The tumor size was measured using the standard formula: 0.54×L×W^2^, L is the longest diameter and W is the shortest diameter. The mice were harvested at day 21.

20(S)-Ginsenoside Rg3 were purchased from MCE (Shanghai, China) and dissolved in saline at the final concentration of 5 mg/mL or 10 mg/mL. Drug treatment began from day 6 after injection of LLC cells.

### 2.3 Cell culture

H1975, H1299, A549 and HCC827 were purchased from Cell Bank of Chinese Academy of Sciences (Shanghai, China). Jurkat cell were purchased from Pricella (Wuhan, China). LLC cell lines were kindly gifted by Prof. Yin Lu in Nanjing university of Chinese medicine. A549 cells were cultured in Ham’s F-12K medium containing 10% fetal bovine serum (FBS). Jurkat, H1975, H1299 and HCC8227 were cultured in RPMI-1640 medium containing 10% FBS. LLC cells were cultured in Dulbecco’s Modified Eagle’s Medium (DMEM) containing 10% FBS. All cells were cultured at a humidified 37°C, 5% CO_2_ incubator.

### 2.4 Western blot

Cells and tissues were lysed using lysis buffer (P0013B, Beyotime) containing a protease inhibitor cocktail (ST506, Beyotime) and a phosphatase inhibitor cocktail (GB-0032, KeyGEN) in a low-temperature freeze grinder. The proteins were denatured, separated by SDS-PAGE, and then transferred onto PVDF membranes (Millipore, USA). Subsequently, the samples were probed with antibodies overnight at 4 °C. After labeling with the primary antibody and Goat Anti-Rabbit IgG (H+L) HRP antibodies (Cat No. BS13278, Bioworld Technology), images were acquired using a BIORAD imaging system (chemiDOCTMXRS, Bio-Rad). The primary antibodies used were as follows: EGFR (Cat No. A23381, 1:1000, Abclonal), Phospho-EGFR-Y1068(Cat No. AP0301, 1:1000, Abclonal), GSK3β (Cat No.9315, 1:1000, Cell Signaling Technology), phospho-GSK3β Ser9 (Cat No.9336, 1:1000, Cell Signaling Technology), PD-L1 (Cat No. A1645, 1:1000, Abclonal) and β-actin (Cat No. AC038, 1:5000, Abclonal).

### 2.5 Real-time quantitative PCR analysis

Cells were lysed in TRIzol (Thermo Fisher, USA), and RNA was isolated using the chloroform-isopropanol method. cDNA was synthesized with the iScript cDNA Synthesis Kit (Yeasen, China) from 500 ng total RNA utilizing HiscriptII QRT SuperMix (Yeasen, China). Real-time PCR was conducted using ChamQ SYBR qPCR Master Mix (Low ROX Premixed) (Yeasen, China) and detected with the ABI 7500 system (Applied Biosystems, CA, USA). The primer sequences were as follows:

CD274 F: 5′-GCTGCACTAATTGTCTATTGGGA-3′

CD274 R: 3′-AATTCGCTTGTAGTCGGCACC-5′

GAPDH F: 5′-GGAGCGAGATCCCTCCAAAAT-3′

GAPDH R: 3′-GGCTGTTGTCATACTTCTCATGG-5′

### 2.6 Tumor cell-T cell coculture

Coculture experiments were conducted as follows. Briefly, 5×10^5 H1975 cells were seeded into 6-well plates overnight. Then 5×10^6 Jurkat T cells were added into cell culture media and incubated for another 12 h. After 12 h, cells were harvested and centrifuged at 500 g for 5 min, followed by resuspended with PBS for further analysis.

### 2.7 Flow cytometry

To determine the effects of Rg3 on IFN-γ induced PD-L1 model, cells were treated with 10 ng/mL IFN-γ with or without 50 μM Rg3 for 24 h. Cells were harvested and stained with PE anti-human CD274 (B7-H1, PD-L1) Antibody (Cat No.393608, Biolegend) at room temperature for 30 min. Cells were then washed with PBS and resuspended in 500 μL PBS for flow analysis.

To detect the activation and cytotoxicity of Jurkat cells, tumor cells and T cells were cocultured as previously described and treated with Rg3 for 24 h. Cells were then harvested, washed and stained with APC anti-human CD3 (Cat No. 981012, Biolegend), PE anti-human CD25 (Cat No. 985802, Biolegend) and 7-AAD Viability Staining Solution (Cat No. 420403, Biolegend) at room temperature for 30 min. Cells were then washed with PBS and resuspended in 500 μL PBS for flow analysis.

To detect the activation of T cells *in vivo*, 100 mg tumor tissues were cut into small pieces and lysed in Triple Enzyme solution (Elabscience, Wuhan, China) at 37°C for 2 h, and filtered by 100-μm cell-strainer. Cells were then washed with PBS and centrifuged at 500 g for 5 min, resuspended, and stained with Pacific Blue™ anti-mouse CD45 Antibody (Cat No. 157211, Biolegend), FITC anti-human/mouse Granzyme B Antibody (Cat No. 515403, Biolegend), APC anti-mouse Perforin Antibody (Cat No. 154403, Biolegend), PE anti-mouse CD3 Antibody (Cat No. 100205, Biolegend) and Brilliant Violet 605™ anti-mouse CD8a Antibody (Cat No. 100743, Biolegend) at room temperature for 30 min. Cells were then washed with PBS and resuspended in 500 μL PBS for flow analysis. Data were acquired by BD FACS Celesta and analyzed by FlowJo V10 software.

### 2.8 ELISA

Cytokine concentration in serum was detected using mouse TNF-α, IFN-γ and IL-2 kits (ExCell Bio, suzhou, China) according to the manufacturer’s instructions. The absorbance of each sample was then measured at 450 nm using Synergy H1 Microplate Reader (Bio-Tek).

### 2.9 Statistical analysis

All data are presented as mean ± standard deviation (SD). The data were analyzed using two-tailed Student’s t-test between two groups and one-way analysis of variance followed by Dunnett’s *post hoc* tests when groups were more than two. *p* < 0.05 was considered statistically significant.

## 3 Results

### 3.1 PD-L1 is highly glycosylated in NSCLC cells

**Figure 1:**
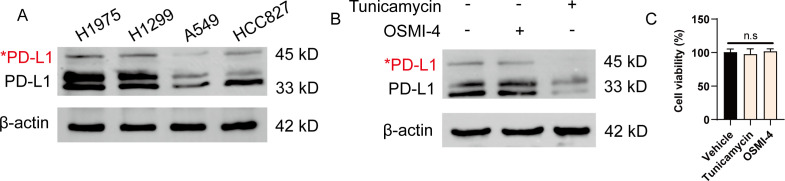
PD-L1 is highly glycosylated in NSCLC cells. **(A)** Western blot analysis of PD-L1 expression in H1975, H1299, A549 and HCC827 cells. Red asterisk indicated the glycosylated form of PD-L1. **(B)** H1975 cells were treated with either 5 μg/mL tunicamycin or 20 μM OSMI-4 for 24 h. The expression of PD-L1 was analyzed by western blot. **(C)** H1975 cells were treated with either 5 μg/mL tunicamycin or 20 μM OSMI-4 for 24 h. Cell viability was determined (n=3). Data were shown as mean ± SD. n.s., not significant.

Previous studies have demonstrated the glycosylation in breast cancer, providing new strategy to target immune checkpoints to enhance anti-tumor immunity (13). To gain better insight into the global glycosylation in lung cancer, we analyzed the glycosylated form of PD-L1 in various cell types, including H1975, H1299, A549 and HCC827. Glycosylation of PD-L1 often results in a heterogeneous pattern on western blots, as we observed for 45-kD band form of PD-L1. Notably, we found that H1975 cells expressed the highest PD-L1 glycosylation among these cells (**Figure 1A**). To further seek out whether *N*-glycosylation or *O*-glycosylation were involved, we utilized specific inhibitors targeting *N*-linked and *O*-linked glycosylation by tunicamycin and OSMI-4, respectively. We found the glycosylation form of PD-L1 was significantly reduced upon tunicamycin treatment, suggesting that PD-L1 is mostly *N*-glycosylated in NSCLC cells (**Figure 1B**). Notably, treatment of these inhibitors did not affect the viability of tumor cells (**Figure 1C**). In conclusion, our data confirm the wide glycosylation of PD-L1 in NSCLC.

### 3.2 Ginsenoside Rg3 inhibited the glycosylation of PD-L1

**Figure 2:**
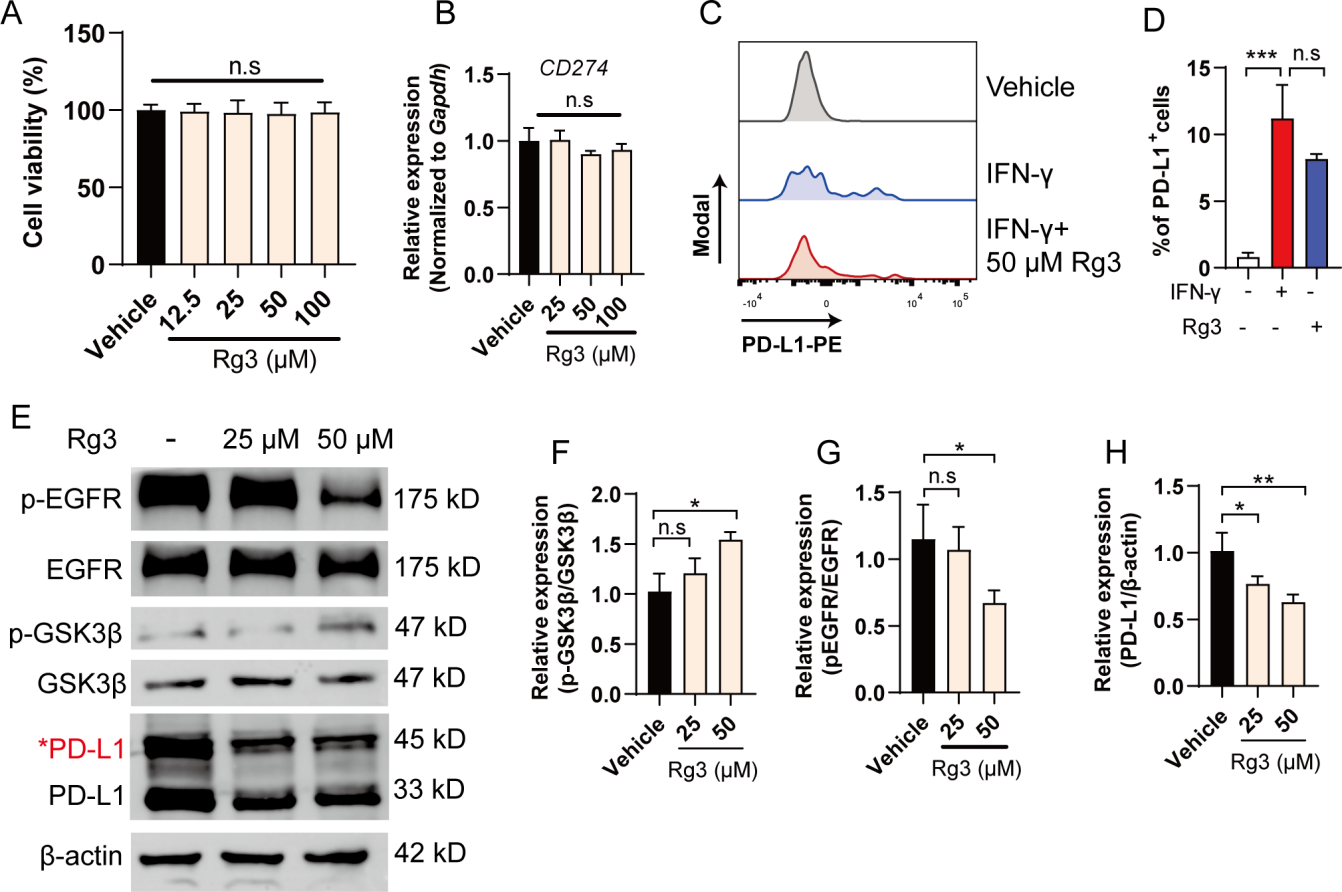
Ginsenoside Rg3 attenuates the glycosylation of PD-L1 through inhibiting EGFR signaling. **(A)** Cell viability (n=3). **(B)** Relative expression of *CD274* after 24 h of Rg3 treatments at indicated concentration (n=3). **(C)** H1975 cells were treated with 10 ng/mL IFN-γ with or without 50 μM Rg3. The percentage of PD-L1^+^cells was determined by flow cytometry. **(D)** Quantification of PD-L1^+^cells (n=3). **(E–H)** H1975 cells were treated with 25 μM or 50 μM Rg3 for 24 h. The expression of p-EGFR, EGFR, p-GSK3β, GSK3β and PD-L1 were determined by western blot. Quantified results were shown. Data were shown as mean ± SD. *p < 0.05, ***p <0.001 compared to vehicle group. n.s., not significant.

Ginsenoside Rg3 exerts anti-tumor responses, confirming the role of Rg3 in treating cancers, yet the mechanisms remain elusive (21–23). This prompts us to further investigate the potential mechanisms underlying anti-tumor effects of Rg3. As H1975 cells exhibited the highest glycosylation, we chose H1975 for further investigation. Of interest, when we treated H1975 with different concentration of Rg3, we did not observe significant inhibition on tumor growth, arguing the notion that Rg3 directly kill tumor cells (**Figure 2A**). To test whether Rg3 could affect PD-L1 translation, we also did not observe the decrease of mRNA expression of PD-L1 (**Figure 2B**). Further, we tested whether Rg3 could influence the expression of PD-L1 upon classical stimulus like IFN-γ. Consistently, IFN-γ markedly upregulated PD-L1 expression in H1975 cells, while Rg3 had no effects on this (**Figures 2C, D**). Thus, we hypothesized that Rg3 might have effect on the post-translational modification on PD-L1. As expected, 50 μM Rg3 significantly reduced the 45-kD band of PD-L1 and 33-kD band of PD-L1, suggesting that Rg3 inhibited the glycosylation of PD-L1 (**Figure 2E**). We also found Rg3 inhibited the glycosylation of PD-L1 in H1299 cells, which also exhibit high level of glycosylated PD-L1 (**Supplementary Figure S1A**).To address how Rg3 affected PD-L1 glycosylation, we noticed that previous researches identified EGFR-GSK3β signaling controls the glycosylation in breast cancer cells (13). The phosphorylation of EGFR induces the inhibitory signal to inactivate GSK3β, which results in less degradation of PD-L1. Interestingly, we found that Rg3 significantly inhibited the phosphorylation of EGFR and the level of p-GSK3β was upregulated (**Figures 2E–H**), demonstrating Rg3 could inactivate EGFR signaling to reduce PD-L1 glycosylation. We further screened some of the enzymes involved in PD-L1 glycosylation, including MGAT3, B3GNT3, GNPTAB, ST6GAL1, MAN2B1, and MGAT5. Interestingly, we found Rg3 could downregulate the expression of *B3GNT3* and *MGAT5*, suggesting that Rg3 might function through these glycotransferases (**Supplementary Figure S2**).

### 3.3 Inhibition of PD-L1 glycosylation enhanced T cell mediated tumor cell killing *in vitro*

**Figure 3:**
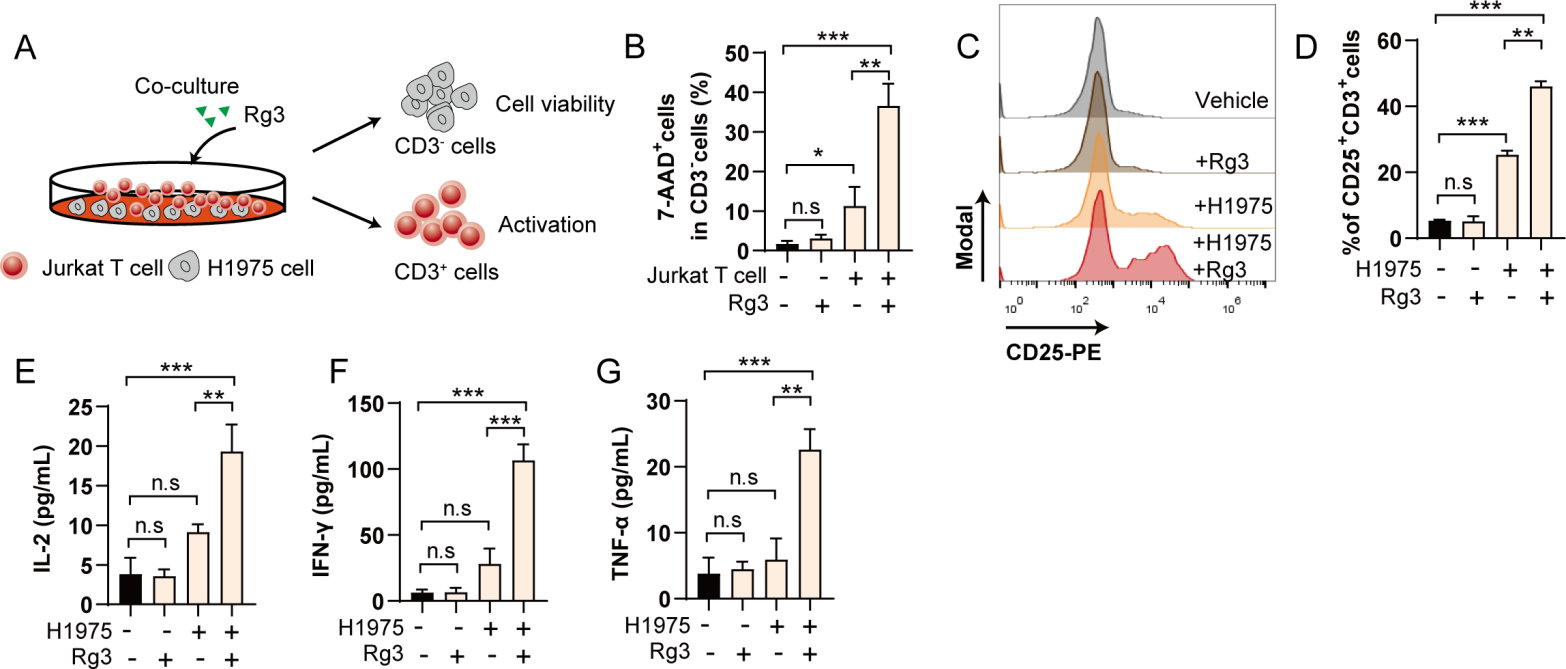
Inhibition of PD-L1 glycosylation enhances T cell mediated tumor cell killing *in vitro*. **(A)** Schematic illustration of coculture experiment. **(B)** Percentage of 7-AAD^+^cells in CD3^-^cells (n=3). **(C)** Histogram of CD25 after coculture and Rg3 treatment. **(D)** Percentage of CD25^+^CD3^+^cells in each group (n=3). **(E–G)** Concentration of IL-2 **(E)**, IFN-γ **(F)** and TNF-α **(G)** in cell culture supernatant (n=3). Data were shown as mean ± SD. *p < 0.05, **p <0.01, ***p <0.001. n.s., not significant.

Having observed the effects of Rg3 on PD-L1 glycosylation, we next wonder whether the inhibition of PD-L1 glycosylation could awaken the anti-tumor responses. To test this hypothesis, we conducted tumor cell-jurkat T cell coculture experiment, the cytotoxicity and activation of T cells were analyzed by flow cytometry (**Figure 3A**). We found that Rg3 alone did not affect the viability of tumor cells, consistent with previous results. Rg3 treatment alone also did not affected the activation of T cells. However, during the coculture experiment, we observed that Rg3 could markedly increase the killing ability of T cells, resulting more 7-AAD^+^cells dead cells (**Figure 3B**), as well as in H1299 cells (**Supplementary Figure S1B**). Moreover, we noticed that the activation of T cells was increased upon Rg3 treatment, as evidenced by the increased population of CD25^+^CD3^+^cells (**Figures 3C, D**). We also found elevated concentration of IL-2, IFN-γ and TNF-α in cell culture supernatants (**Figures 3E–G**). These results confirm that Rg3 could activate T cells when cultured with tumor cells, further indicating the immunoregulatory role of Rg3.

### 3.4 Ginsenoside Rg3 inhibited tumor cell growth and attenuated PD-L1 glycosylation *in vivo*

**Figure 4:**
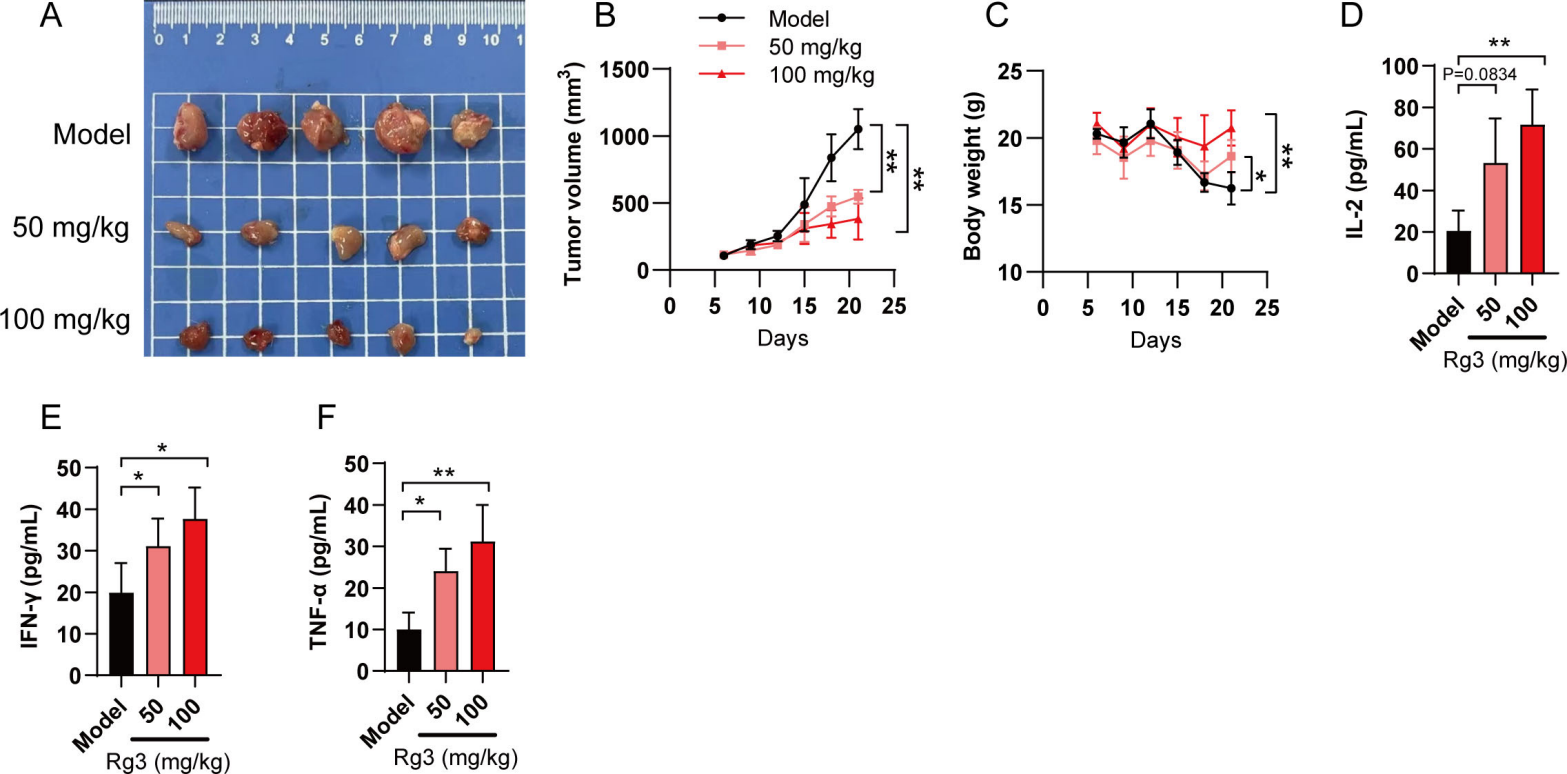
Ginsenoside Rg3 inhibits tumor cell growth *in vivo*. **(A)** Mice were transplanted with LLC cells and orally administered indicated concentration of Rg3. 21 days after tumor cell inoculation, tumors were harvested and representative images of tumors in each group were shown (n=5). **(B)** Tumor volume in each group (n=5). **(C)** Body weight. **(D–F)** Serum concentration of IL-2 **(D)**, IFN-γ **(E)** and TNF-α **(F)** in each group (n=5). Data were shown as mean ± SD. *p < 0.05, **p <0.01 compared to model group.

Since we confirmed the anti-tumor response of Rg3 was primarily mediated by inhibiting PD-L1 glycosylation and subsequently enhancing immunity, we further tested the anti-tumor responses of Rg3 in immunocompetent mice. LLC xenograft model was used to assess whether Rg3 could inhibit tumor growth *in vivo*. LLC cells also have prominent glycosylated form of PD-L1 (**Supplementary Figure S3**). As expected, we found that Rg3 inhibited tumor cell growth in a dose-dependent manner, as both 50 mg/kg and 100 mg/kg Rg3 could inhibit tumor volume and increase the body weight (**Figures 4A–C**). Moreover, we found elevated serum IL-2, IFN-γ and TNF-α in Rg3 treated mice, suggesting that Rg3 treatment could enhance the activation of T cells *in vivo* (**Figures 4D–F**).

**Figure 5:**
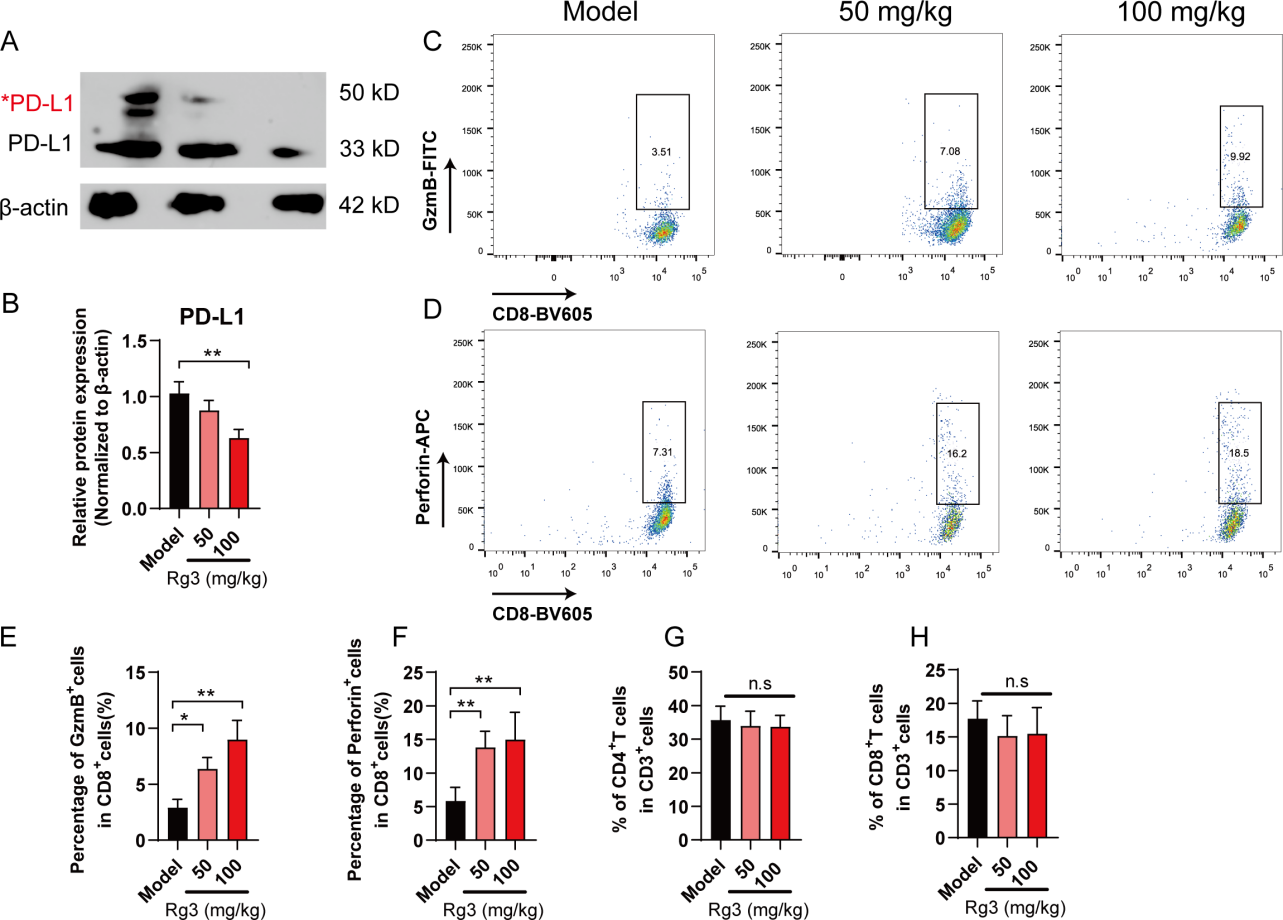
Ginsenoside Rg3 attenuated PD-L1 glycosylation and restored anti-tumor immunity. **(A)** Western blot analysis of PD-L1 in tumor tissues. Red asterisk indicated the glycosylated form of PD-L1. **(B)** Relative PD-L1 protein expression (n=3). **(C–F)** Flow cytometry analysis of GzmB^+^ **(C, E)** and Perforin^+^ **(D, F)** CD8^+^T cells in tumor microenvironment (n=5). **(G)** Percentage of CD4^+^T cells in tumor microenvironment (n=5). **(H)** Percentage of CD8^+^T cells in tumor microenvironment (n=5). Data were shown as mean ± SD. *p < 0.05, **p <0.01 compared to model group.

To test whether Rg3 could inhibit PD-L1 glycosylation *in vivo*, we analyzed the expression of PD-L1 in tumor tissues. As expected, the glycosylation of PD-L1 was dramatically decreased in Rg3 group (**Figures 5A, B**). To further confirm the role of Rg3 in CD8^+^T cell activation, we analyzed the expression of perforin and granzyme B (GzmB) in CD8^+^T cells in tumor microenvironment. Rg3 treatment did not alter the composition of CD4/CD8 T cells, but we found that both 50 mg/kg and 100 mg/kg Rg3 could promote the expression of perforin and GzmB, which represented the activation of CD8^+^T cells (**Figures 5C–H**). Collectively, our data demonstrated Rg3 could inhibit the glycosylation of PD-L1 and enhance anti-tumor immunity.

## 4 Discussion

Multiple transcription factors such as STAT, ATF3, c-Myc and NF-κB are novel regulators of PD-L1, yet the post-translational modification of PD-L1 is also of great importance in understanding the potential mechanism of immune escape (24, 25). Here, we identified that PD-L1 is commonly glycosylated in NSCLC cell lines. Notably, other studies confirmed the existence of PD-L1 glycosylation in human tumor samples, prompting us to investigate the potential treatment against glycosylation. There are two types of protein glycosylation, including *N*-linked and *O*-linked glycosylation. *N*-linked glycosylation of the extracellular domain of PD-L1 could promote the stability by inhibiting 26S proteosome-dependent degradation, while *O-*linked β-N-acetylglucosamine modification promotes tumor immune evasion by inhibiting PD-L1 lysosomal degradation (16). By utilizing different inhibitors, we demonstrated that PD-L1 is mostly *N*-linked glycosylation in NSCLC cells.

Ginsenoside Rg3 is mainly derived from the Chinese herb Panax ginseng Meyer, have been extensively studied and is well-known for its immune regulatory effects (26, 27). In this study, we uncovered new mechanism of Rg3 in regulating anti-tumor immunity, that is, inhibiting the glycosylation of PD-L1. Notably, EGF has been proven to upregulate PD-L1 glycosylation through EGFR signaling, which can upregulate B3GNT3 glycosyltransferase to mediate PD-L1 glycosylation, further inhibit the activity of GSK3β, thus impair the degradation of PD-L1 (28). Our research demonstrated that Rg3 inhibited the phosphorylation of EGFR, accompanied by downregulating the expression of p-GSK3β, thus reduced the expression of PD-L1. Further, we confirmed the enhanced anti-tumor immunity in tumor cell-T cell coculture system upon Rg3 treatment, suggesting that Rg3 did not directly kill tumor cells, instead, could strengthen the function of T cells by inhibiting the PD-1/PD-L1 axis.

Finally, we elucidated the anti-tumor effects of Rg3 in a mouse xenograft model. Rg3 could significantly promote the cytotoxicity of CD8^+^T cells in microenvironment, thus lead to the reduction of tumor volume. Our research can provide new insight into the development of immunotherapies, and the combination of Rg3 and other classical drugs or immune cell-based therapies may be beneficial to NSCLC and even other cancer patients.

In summary, our research demonstrated that ginsenoside Rg3 could significantly inhibit the glycosylation of PD-L1, thus enhance the T cell-mediated anti-tumor immunity. Rg3 may be potential drugs against NSCLC and the combination of Rg3 and other targeted therapies may provide new therapeutical strategy in treating NSCLC.

## Data Availability

The original contributions presented in the study are included in the article/**Supplementary Material**. Further inquiries can be directed to the corresponding authors.
