# Supplementary material for: Ginsenoside Rg3 targets glycosylation of PD-L1 to enhance anti-tumor immunity in non-small cell lung cancer
Source: Front Immunol. 2024 Aug 23;15:1434078. doi: 10.3389/fimmu.2024.1434078 (PMC11377313; doi:10.3389/fimmu.2024.1434078)
Supplement: Supplementary file 1 [file DataSheet1.pdf]

Supplementary Figures

Figure S1

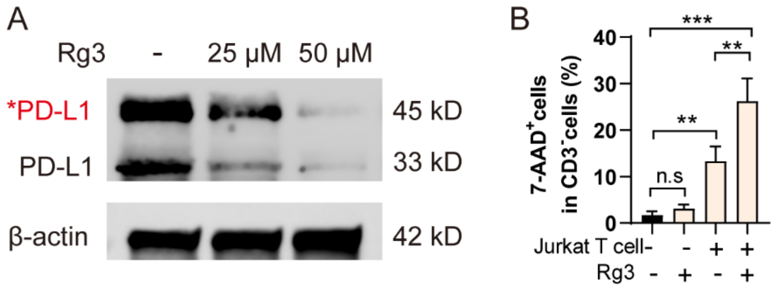

Figure S1. Ginsenoside Rg3 attenuates the glycosylation of PD-L1 in H1299 cells. (A) H1299 cells were treated with 25 μM or 50 μM Rg3 for 24 h. The expression of PD-L1 were determined by western blot. (B) Percentage of 7-AAD^{+}cells in CD3^{-}cells (n=3).

Figure S2

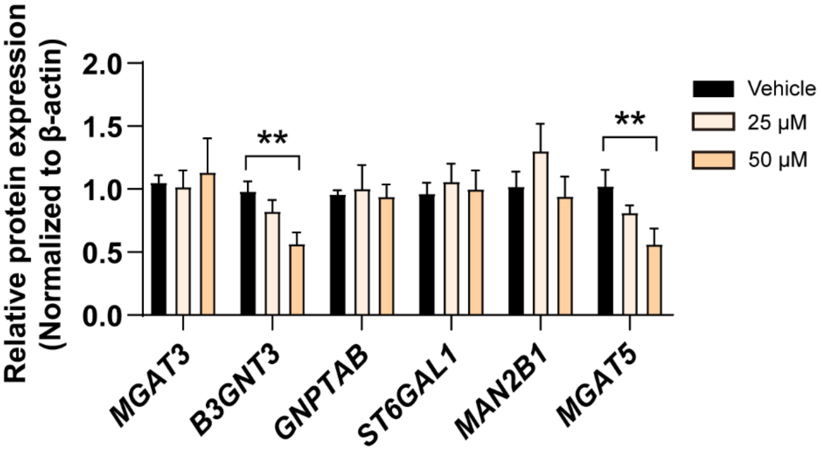

Figure S2. Rg3 inhibited glycotransferases. H1975 cells were treated with 25 μM or 50 μM Rg3 for 24 h. The expression of MGAT3, B3GNT3, GNPTAB, ST6GAL1, MAN2B1 and MGAT5 were analyzed by qPCR (n=3). Data were shown as mean ± SD. **p <0.01 compared to vehicle group.

Figure S3

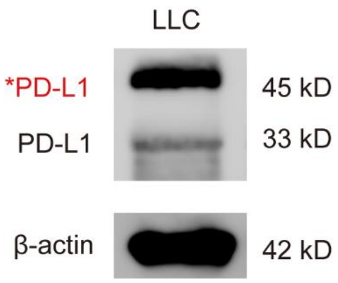

Figure S3. Expression of PD-L1 in LLC cells.
